# NeuroLINCS Proteomics: Defining human-derived iPSC proteomes and protein signatures of pluripotency

**DOI:** 10.1038/s41597-022-01687-7

**Published:** 2023-01-11

**Authors:** Andrea D. Matlock, Vineet Vaibhav, Ronald Holewinski, Vidya Venkatraman, Victoria Dardov, Danica-Mae Manalo, Brandon Shelley, Loren Ornelas, Maria Banuelos, Berhan Mandefro, Renan Escalante-Chong, Jonathan Li, Steve Finkbeiner, Ernest Fraenkel, Jeffrey Rothstein, Leslie Thompson, Dhruv Sareen, Clive N. Svendsen, NIH NeuroLINCS Consortium, Jennifer E. Van Eyk, Ritchie Ho, Brook Wassie, Natasha Patel-Murray, Pamela Milani, Miriam Adam, Karen Sachs, Alex Lenail, Divya Ramamoorthy, Gavin Daigle, Uzma Hussain, Julia Kaye, Leandro Lima, Jaslin Kalra, Alyssa Coyne, Ryan G Lim, Jie Wu, Jennifer Stocksdale, Terri G Thompson, Jennifer E. Van Eyk

**Affiliations:** 1 NeuroLINCS, Advanced Clinical Biosystems Research Institute, Cedars-Sinai Medical Center, Los Angeles, CA 90048 USA; 2 NeuroLINCS, Regenerative Medicine Institute, Cedars-Sinai Medical Center, Los Angeles, CA 90048 USA; 3 NeuroLINCS, Department of Biological Engineering, MIT, Cambridge, MA 02142 USA; 4 NeuroLINCS, Gladstone Institute of Neurological Disease and the Departments of Neurology and Physiology, University of California San Francisco, San Francisco, CA 94158 USA; 5 NeuroLINCS, Department of Neuroscience, Johns Hopkins University, Baltimore, MD 21205 USA; 6 NeuroLINCS, Departments of Psychiatry and Human Behaviour, Neurobiology and Behaviour and UCI MIND, University of California Irvine, Irvine, CA 92697 USA

**Keywords:** Induced pluripotent stem cells, Amyotrophic lateral sclerosis, Mass spectrometry, Predictive markers

## Abstract

The National Institute of Health (NIH) Library of integrated network-based cellular signatures (LINCS) program is premised on the generation of a publicly available data resource of cell-based biochemical responses or “signatures” to genetic or environmental perturbations. NeuroLINCS uses human inducible pluripotent stem cells (hiPSCs), derived from patients and healthy controls, and differentiated into motor neuron cell cultures. This multi-laboratory effort strives to establish i) robust multi-omic workflows for hiPSC and differentiated neuronal cultures, ii) public annotated data sets and iii) relevant and targetable biological pathways of spinal muscular atrophy (SMA) and amyotrophic lateral sclerosis (ALS). Here, we focus on the proteomics and the quality of the developed workflow of hiPSC lines from 6 individuals, though epigenomics and transcriptomics data are also publicly available. Known and commonly used markers representing 73 proteins were reproducibly quantified with consistent expression levels across all hiPSC lines. Data quality assessments, data levels and metadata of all 6 genetically diverse human iPSCs analysed by DIA-MS are parsable and available as a high-quality resource to the public.

## Abstract (summary)

**Table:** 

Measurement(s)	Protein expression
Technology Type(s)	Mass Spectrometry
Factor Type(s)	genetically distinct human cell lines
Sample Characteristic - Organism	Homo sapiens
Sample Characteristic - Location	United States

## Background & Summary

NeuroLINCS (http://neurolincs.org/), is one of several data generation centers of the National Institute of Health (NIH) Library of integrated network-based cellular signature (LINCS)^1^. It is comprised of a collaboration across seven specialized laboratories to support the multi-omic data generation and data integration initiatives for the motor neuron disorders amyotrophic lateral sclerosis (ALS) and spinal muscular atrophy (SMA)^2^. All ALS cell lines analysed originate from a subset of ALS patients with genetic mutations in C9orf72 (C9), superoxide dismutase 1 (SOD1) or were derived from sporadic disease^3–6^. SMA cell lines contain various genetic mutations in SMN1 that reduce expression and reduce or inhibit normal protein function. SMA patients are often diagnosed in early childhood whereas ALS is diagnosed later in life, 55 being the average age of onset.

Biomolecular studies of human neurological disorders have transitioned to hiPSC differentiated cell types affected in diseases since human tissue samples or biopsies can only be obtained posthumously and thus less informative for studies of disease progression. NeuroLINCS focuses on hiPSCs differentiated to motor neuron cell cultures. The biological interrogation of human neuronal cultures is possible due to advances in hiPSC line generation^7^ and neuronal differentiation protocols, as long as the culture protocol is consistent^8–14^.

**Fig. 1:**
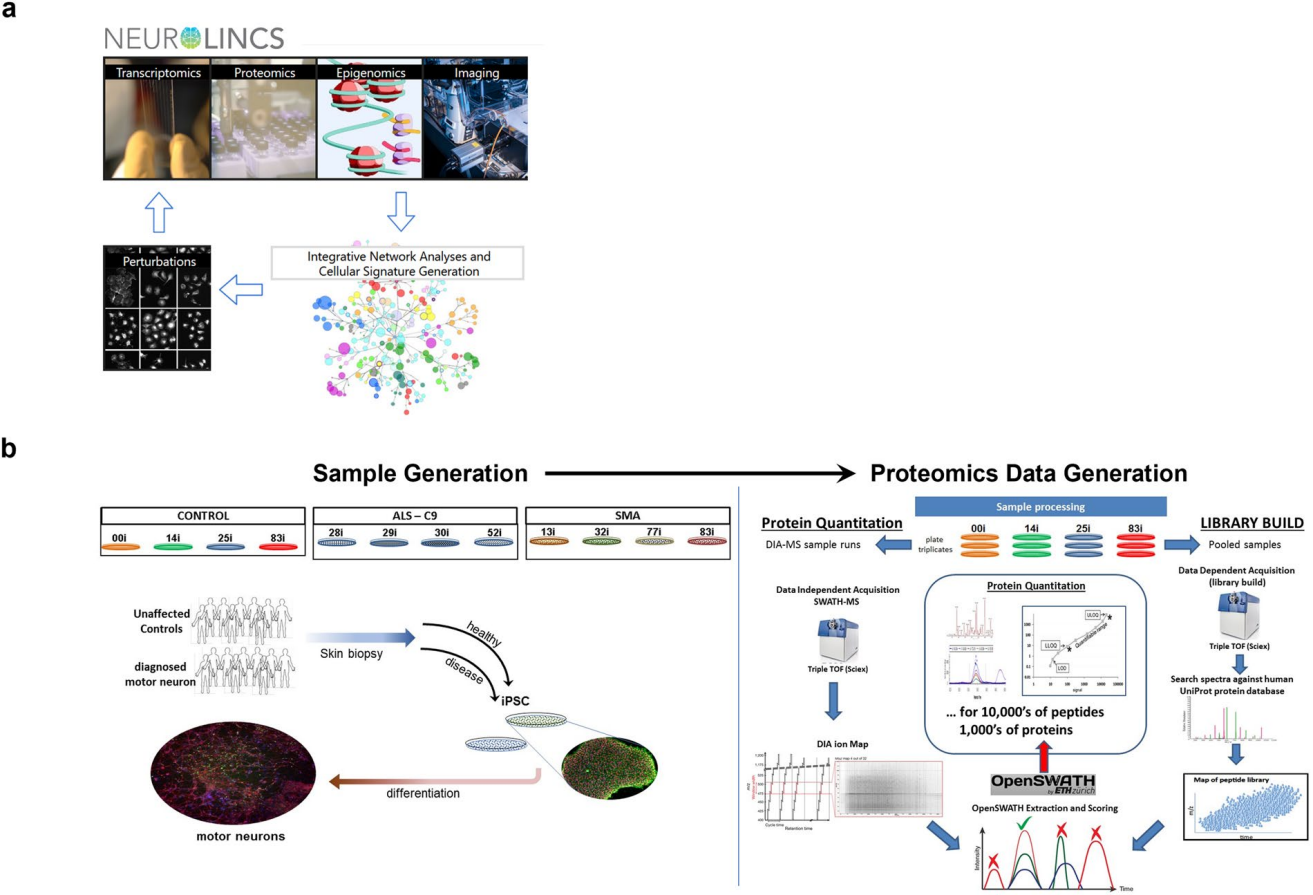
NeuroLINCS project overview. (**a**) Omic data generation centers include transcriptomics, proteomics, epigenomics, robotic imaging assays and cell-based functional studies. Molecular signatures of disease from epigenetic, RNA and protein analyses are incorporated into integrative network analysis using Omic Integrator. (**b**) Samples generated from 12 patient-derived hiPSC lines. Proteomics data is generated using DIA-MS methods on TripleTOFs (Sciex) and searched using sample specific peptide spectral libraries generated from DIA-MS of pooled samples. Analyte signals are extracted using OpenSWATH and MapDIA as specified.

The goal of NeuroLINCS is to generate and combine multi-omic data sets to produce weighted disease signatures from epigenomic, transcriptomic and proteomic pathway analyses from the same live cell specimens to test functional phenotypes using specialized robotic imaging assays^15–17^, and functional assays (Fig. 1a)^18–20^. Of the twelve cell lines (fig 1b), six lines were selected, two per biological condition: patients with the C9orf72 ALS mutation (C9)^21^, patients with SMA^22^, and heathy or undiagnosed motor neuron controls (Fig. 1b). hiPSCs and motor neuron cultures were prepared in duplicate or triplicate at Cedars-Sinai Stem Cell Core (https://www.cedars-sinai.org/research/areas/biomanufacturing/ipsc.html). To assess the challenges around carrying out proteomics on hiPSC and derived motor neurons, it was important to document the number of times a cell line did not grow appropriately, or cells numbers were insufficient for adequate proteome depth. It is equally important to understand the stability of the differentiation protocol and the data generation workflow over time by analysing i) biological growth replicates for which the same hiPSC cell line was collected from different wells but cultured simultaneously and ii) sample data re-collection from a digested sample to ensure the stability of sample storage and the performance of the mass spectrometer over the course of months.

A summary of all NeuroLINCS hiPSC and differentiated motor neuron data publicly released through the NIH LINCS program^23,24^ are provided (Table 1). Data-independent acquisition-mass spectrometry (DIA-MS) is a quantitative discovery tool in proteomics and its application has gained recognition in clinical biomarker analyses^25–27^. NeuroLINCS proteomic data was generated using SWATH-MS, a data-independent acquisition-mass spectrometry (DIA-MS) method^28^, to reproduciably quantify approximately 3,000 proteins in every motor neuron sample of the NeuroLINCS project.

**Table 1:** NeuroLINCS data publicly released. A, available; n/a, not available.

Cell type	Cell Line	Diagnosis	Differentiaion protocol	ATAC-Seq			RNA-Seq			DIA-MS Proteomics			
				Leve l	Leve 3	Leve 4	Leve 1	Leve 3	Leve 4	Leve 0	Leve 2	Leve 3	Leve 4
**iPSC**	CS14iCTR-n6	Control	none	A	A	A	A	A	A	A	A	A	A
	CS25iCTR-18n2	Control	none	A	A	A	A	A	A	A	A	A	A
	CS83iCTR-33n1	Control	none	A	A	A	A	A	A	A	A	A	A
	CS28iALS-n2A	ALS - C9orf72	none	A	A	A	A	A	A	A	A	A	A
	CS29iALS-n1N	ALS - C9orf72	none	A	A	A	A	A	A	A	A	A	A
	CS30iALS-n1A	ALS - C9orf72	none	A	A	A	A	A	A	A	A	A	A
	CS52iALS-n6A	ALS - C9orf72	none	n/a	n/a	n/a	A	A	A	A	A	A	A
	CS32iSMA-n3	SMA Type I	none	A	A	A	A	A	A	A	A	A	A
	CS77iSMA-n5	SMA Type I	none	A	A	A	A	A	A	A	A	A	A
	CS83iSMA-n5	SMA Type I	none	A	A	A	A	A	A	A	A	A	A
**iMNs**	CS00iCTR_iMNS	Control	Long	A	A	A	A	A	A	A	A	A	A
	CS25iCTR_iMNS	Control	Long	A	A	A	A	A	A	A	A	A	A
	CS83iCTR_iMNS	Control	Long	A	A	A	A	A	A	A	A	A	A
	CS28iALS_iMNS	ALS - C9orf72	Long	A	A	A	A	A	A	A	A	A	A
	CS29iALS_iMNS	ALS - C9orf72	Long	A	A	A	A	A	A	A	A	A	A
	CS30iALS_iMNS	ALS - C9orf72	Long	A	A	A	A	A	A	A	A	A	A
	CS52iALS_iMNS	ALS - C9orf72	Long	A	A	A	A	A	A	A	A	A	A
	CS32iSMA_iMNS	SMA Type I	Long	A	A	A	A	A	A	A	A	A	A
	CS77iSMA_iMNS	SMA Type I	Long	A	A	A	A	A	A	A	A	A	A
	CS83iSMA_iMNS	SMA Type I	Long	A	A	A	A	A	A	A	A	A	A
**diMNs**	CS00iCTR_diMNS	Control	Short	A	A	A	A	A	A	A	A	A	A
	CS14iCTR_diMNS	Control	Short	A	A	A	A	A	A	A	A	A	A
	CS25iCTR_diMNS	Control	Short	A	A	A	A	A	A	n/a	n/a	n/a	n/a
	CS83iCTR_diMNS	Control	Short	A	A	A	A	A	A	n/a	n/a	n/a	n/a
	CS04iALS_diMNS	ALS - SOD1	Short	A	A	A	A	A	A	A	A	A	A
	CS11iALS_diMNS	ALS - SOD1	Short	A	A	A	A	A	A	A	A	A	A
	CS14iALS_diMNS	ALS - SOD1	Short	A	A	A	A	A	A	A	A	A	A
	CS22iALS_diMNS	ALS - SOD1	Short	A	A	A	A	A	A	A	A	A	A
	CS28iALS_diMNS	ALS - C9orf72	Short	A	A	A	A	A	A	A	A	A	A
	CS29iALS_diMNS	ALS - C9orf72	Short	A	A	A	A	A	A	A	A	A	A
	CS30iALS_diMNS	ALS - C9orf72	Short	A	A	A	A	A	A	A	A	A	A
	CS52iALS_diMNS	ALS - C9orf72	Short	A	A	A	A	A	A	A	A	A	A
	CS138isALS_diMNS	ALS - Sporadic	Short	A	A	A	A	A	A	A	A	A	A
	CS152isALS_diMNS	ALS - Sporadic	Short	A	A	A	A	A	A	A	A	A	A
	CS166iALS_diMNS	ALS - Sporadic	Short	A	A	A	A	A	A	A	A	A	A
	CS32iSMA_diMNS	SMA Type I	Short	n/a	n/a	n/a	n/a	n/a	n/a	n/a	n/a	n/a	n/a
	CS77iSMA_diMNS	SMA Type I	Short	n/a	n/a	n/a	n/a	n/a	n/a	n/a	n/a	n/a	n/a
	CS83iSMA_diMNS	SMA Type I	Short	n/a	n/a	n/a	n/a	n/a	n/a	n/a	n/a	n/a	n/a

Aligned with the vision and goals of the NIH LINCS program, to make a long-standing data resource to the public, data quality assessments and supporting metadata are available for overall transparency. The NIH Data integration and coordination center (DCIC) introduced a system to designate and provide access to each respective data level with the intention of enabling broad applicability throughout the scientific community^1^. The levels have been organized to be consistent across various assay types and LINCS data generation centers. These include, raw data files, unprocessed/pre-normalized protein and peptide relative quantitation values, normalized protein/peptide quantitation, and biological signatures. This format inherently provides perspective for potential data users to the relevant data level applicable to their interest and expertise. For example, data level 0 contain raw MS data files for remining peptide spectra or testing novel mass spectral computational tools. The last data level containing biological signatures for NeuroLINCS contains hiPSC protein markers and differential protein expression signals. Neurobiologists looking for a specific protein or peptide of interest may parse through disease specific protein signatures compiled in the final processed data level. It should go without saying, any publicly available data set requires careful consideration and data quality assessments regardless of data type^29–35^. Many previously reported hiPSC protein markers are observed in all hiPSC analysed and are discussed below.

## Methods

### hiPSC lines, culture and maintenance

Fibroblasts from ALS, SMA and control donors were reprogrammed into hiPSCs using nucleofection of episomal plasmids containing POU5F1, SOX2, KLF4, LIN28, L-MYC, TP53shRNA as described in previously published manuscripts^21,36,37^. All the cell lines and protocols in the present study were carried out in accordance with the guidelines approved by Stem Cell Research Oversight committee (SCRO) and Institutional Review Board (IRB) at the Cedars-Sinai Medical Center under the auspice IRB-SCRO Protocols Pro00032834 (iPSC Core Repository and Stem Cell Program). Human iPSCs were cultured in mTeSR®1 medium (StemCell Technologies, Cat. 85850) on growth factor-reduced Matrigel™ Matrix (Corning, Cat. 354230) coated plates at 37 °C in a 20% O_2_, 5% CO_2_ incubator. Briefly, 70–90% confluent hiPSC colonies were passaged every 7 days chemically (Versene, Life Technologies, Cat. 15040-066) or mechanically by StemPro® EZPassage™ Disposable Stem Cell Passaging Tool (Life Technologies, Cat. 23181–010). The hiPSCs in this study were passaged every 5–7 days. The hiPSCs were cryopreserved using CryoStor CS10 (StemCell Technologies, Cat. 07930) and an isopropanol freezing vessel at −80 °C overnight. The cryopreserved vials were subsequently stored in liquid nitrogen tanks for long-term storage. Within the various samples produced for proteomics analysis, there were biological growth replicates in which the same hiPSC line was collected from different wells but cultured simultaneously and each well was processed for mass spectral analysis as independent, biological replicate samples.

### Sample preparation

Cell pellets were lysed in 2% SDS, 0.1% TCEP and sonicated for 30 minutes at 70 amp, 10 second on/off pulses (QSonica Q800R) before transfer to 30 kD MWCO filters according to the FASP sample processing protocol^38^. SDS was removed by buffer exchange with 8 M urea into Tris, pH 8 and samples were alkylated using iodoacetamide. Protein digestion was performed in 50 mM NH_4_HCO_3_, pH 8, with Trypsin/LysC mix (Promega) overnight while shaking at 37 °C. Digested sample was desalted and cleaned for mass spectral analysis using Oasis MCX 96-well plates (Waters) and resulting samples were dried and reconstituted in 0.1% FA H_2_0. Liquid Chromatography retention time standards (Biognosys) were added to each sample before analysis by mass spectrometry.

### Mass spectrometry

Human inducible pluripotent stem cells were analysed on the Triple TOF 6600 or 5600 instruments (Sciex)^39^. A sample specific spectral  library was generated from pooled samples of each biological condition, control, ALS and SMA, i.e. control samples were only pooled with controls and 19 DDA-MS analyses were performed. The 19 DDA-MS data files used to make the sample specific spectral library, as well as the spectral library file generated in OpenSWATH are available (https://panoramaweb.org/NeuroLINCS_iPSCs.url and PXD021497)^40^. DIA-MS methods used 100 variable windows over a chromatographic gradient of 120 minutes in the 400–1200 m/z range. Additional experimental metadata are accessible on the NIH LINCS data portal (https://lincsportal.ccs.miami.edu/datasets/)^24^ and available for download (Table 2). Note, data level 0 and 1 are only on panorama web and not available through the NIH portal due to data size limitations.

**Table 2:** Data levels and public access.

**Level 0**	DIA-MS Raw data files (.wiff) DDA-MS library data files (.wiff) hiPSC ion library (TraML)	**Panorama** https://panoramaweb.org/NeuroLINCS_iPSCs.url 10.6069/50qp-cy56 **Proteome Exchange** PXD021497^40^
**Level 1**	Skyline document of 73 quantified hiPSC proteins	**Panorama** https://panoramaweb.org/NeuroLINCS_iPSCs.url^40^
**Level 2**	raw counts (no normalization) for protein and peptides, removal of non-proteotypic peptides, QC	**NIH LINCS data portal**^23,24^ https://lincsportal.ccs.miami.edu/datasets/
**Level 3**	Normalized counts for protein and peptides, removal of non-proteotypic peptides, QC	**NIH LINCS data portal**^23,24^ https://lincsportal.ccs.miami.edu/datasets/
**Level 4**	For hiPSCs (Exp 1): 73 quantified hiPSC proteins. For iMN (Exp 2, 3): Signatures, Fold change (a) peptide level (b) protein levels	**NIH LINCS data portal**^23,24^ https://lincsportal.ccs.miami.edu/datasets/

### Data analysis

Peptide spectral library and data analysis of DIA-MS data were performed as previously described for Triple TOF data^41^. OpenSWATH algorithum^42^ was used for both spectral ion library generation from peptide identification output files generated from DDA^43^ data files and for peptide quantitation from DIA data by extraction of transition ions. Peptide quantitation values are compiled into protein level quantitation using MapDIA v2.4.1^44^ and described in more detail below.

#### Spectral library generation using DDA-MS

Profile-mode.wiff files from the data acquisition were converted to mzML format using the Sciex Data Converter (in proteinpilot mode), version 1.3, and then re-converted to mzXML format using ProteoWizard v.3.0.6002^45^ for peaklist generation. The MS2 spectra were queried against the reviewed canonical Human Uniprot complete proteome database as of July, 2019 appended with iRT protein sequence and shuffled sequence decoys^46^. All data were searched using the X!Tandem Native v.2013.06.15.1, X!Tandem Kscore v.2013.06.15.1^47^ and Comet v.2014.02 rev.2^48^. The search parameters included the following criteria: static modifications of Carbamidomethyl (C) and variable modifications of Oxidation (M). The precursor ion mass tolerance was set to be 50 p.p.m, and mono-isotopic fragment mass tolerance was 100 p.p.m and subsequently filtered to be < 0.05 Da for building spectral library; tryptic peptides with up to three missed cleavages were allowed. The identified peptides were processed and analysed through Trans-Proteomic Pipeline v.4.8^49,50^ and was validated using the PeptideProphet^51^ scoring and the PeptideProphet results were statistically refined using iProphet^52^. All the peptides were filtered at a false discovery rate (FDR) of 1%. The raw spectral libraries were generated from all valid peptide spectrum matches and then refined into the non-redundant consensus libraries^53^ using SpectraST v.4.0^54^. For each peptide, the retention time was mapped into the iRT space^55^ with reference to a linear calibration constructed for each data analyses as by Collins *et al*.^53^. Peptide spectral library was constructed using the top six most intense transitions.

#### Targeted data analysis for DIA-MS

DIA-MS wiff files from the data-independent acquisition were first converted to profile mzML using ProteoWizard v.3.0.6002^45^. The whole process of SWATH-targeted data analysis was carried out using OpenSWATH v.2.0.0^56^ running on an internal computing cluster. OpenSWATH utilizes a target-decoy scoring system (PyProphet v.0.13.3) such as mProphet to estimate the identification of FDR. The best scoring classifier that was built from the sample of most protein identifications was utilized in this study. Based on our final spectral library, OpenSWATH firstly identified the peak groups from all individual SWATH maps at a global peptide FDR of 1% and aligned them between SWATH maps based on the clustering behaviors of retention time in each run with a non-linear alignment algorithm^57^. For this analysis, the MS runs were realigned to each other using Locally Weighted Scatterplot Smoothing method and the peak group clustering was performed using ‘LocalMST’ method. Only peptide peak groups that deviate within 3 standard deviations from the retention time were reported and considered for alignment with the max FDR quality of 5% (quality cutoff to still consider a feature for alignment). To obtain a quantitative data at the protein level, proteins whose peptides were shared between multiple different proteins (non-proteotypic peptides) were discarded from protein level analysis and reporting^57^. This step reduces the total number of peptides and proteins reported even though the peptides are unambiguously identified because it is unclear which or if all the possible protein matches are present in the sample. This step becomes necessary to strengthen the biological pathway analysis of proteins by eliminating inaccurate biological pathways that would result from the inclusion of proteins isoforms or variants identified using peptides shared by multiple proteins. Data pre-processing and statistical analysis of MS runs into quantitative protein data was performed using mapDIA v2.4.1^44^. The transition ion intensities were normalized by total intensity sums as well as a novel alternative normalization by local intensity sums in retention time space to remove systematic bias between MS runs. This is followed by outlier removal and peptide/fragment selection that preserve the major quantitative patterns across all samples for each protein. The selected transition and peptide ions which are unambiguously unique proteotypic peptides assigned to a single specific protein were used in the final model-based statistical significance analysis of protein-level differential expression between specified groups of samples. Quantitative peptide and protein level summary outputs generated by mapDIA v2.4.1^44^ were then used for all downstream biological pathway analyses.

## Data Records

Cell line information and omic assay data, metadata and SOPs can be accessed and downloaded through the NIH LINCS website. Access to all data levels is either through the NIH LINCS data portal (https://lincsportal.ccs.miami.edu/datasets/)^23,24^ or through panorama^40^ (Table 2). Samples, raw file naming and mapping to figure abbreviations and sample replicate metadata are available through the NIH LINCS portal and online Supplementary Table 4. Omic data integration analyses are performed using Omics Integrator on differentiated neuron cultures and is published separately (http://fraenkel-nsf.csbi.mit.edu/omicsintegrator/)^58,59^. The proteomics data released includes the complete pre- and post-normalized peptide and protein lists, a skyline document composed of 73 stable proteins signatures (online Supplementary Table 1)  of hiPSCs and all DIA and DDA raw data files used for this data analysis and to generate sample specific spectral  libraries, respectively (Table 2). In addition, members of the DCIC have developed and given public access to data mining tools available through the NIH LINCS program data portal^23,24^. Shamsaei and Meller, of the BD2K-LINCS DCIC, have contributed several assets to overall LINCS proteomics including a LINCS proteomics website, http://www.lincsproteomics.org/lincsproteomics/ and a novel proteomics peptide data-to-knowledge tool piNET^60^.

## Technical Validation

Technical validation efforts of NeuroLINCS proteomic data include cell line and data quality assessments for the hiPSCs samples and data generated. Cell lines are generated and cultured by the Cedars-Sinai’s Induced Pluripotent Stem Cell Core. Routine quality control assessments are performed during hiPSC line generation, maintenance, banking and experimental use as described above. Out of 12 lines, 2 lines (an SMA line and a control line) were not able to be used after rigorous genomic quality control assessments performed routinely by the iPSC core determined that these cell lines cultures were contaminated with another cell line. Therefore, these samples were dropped from further analysis.

**Fig. 2:**
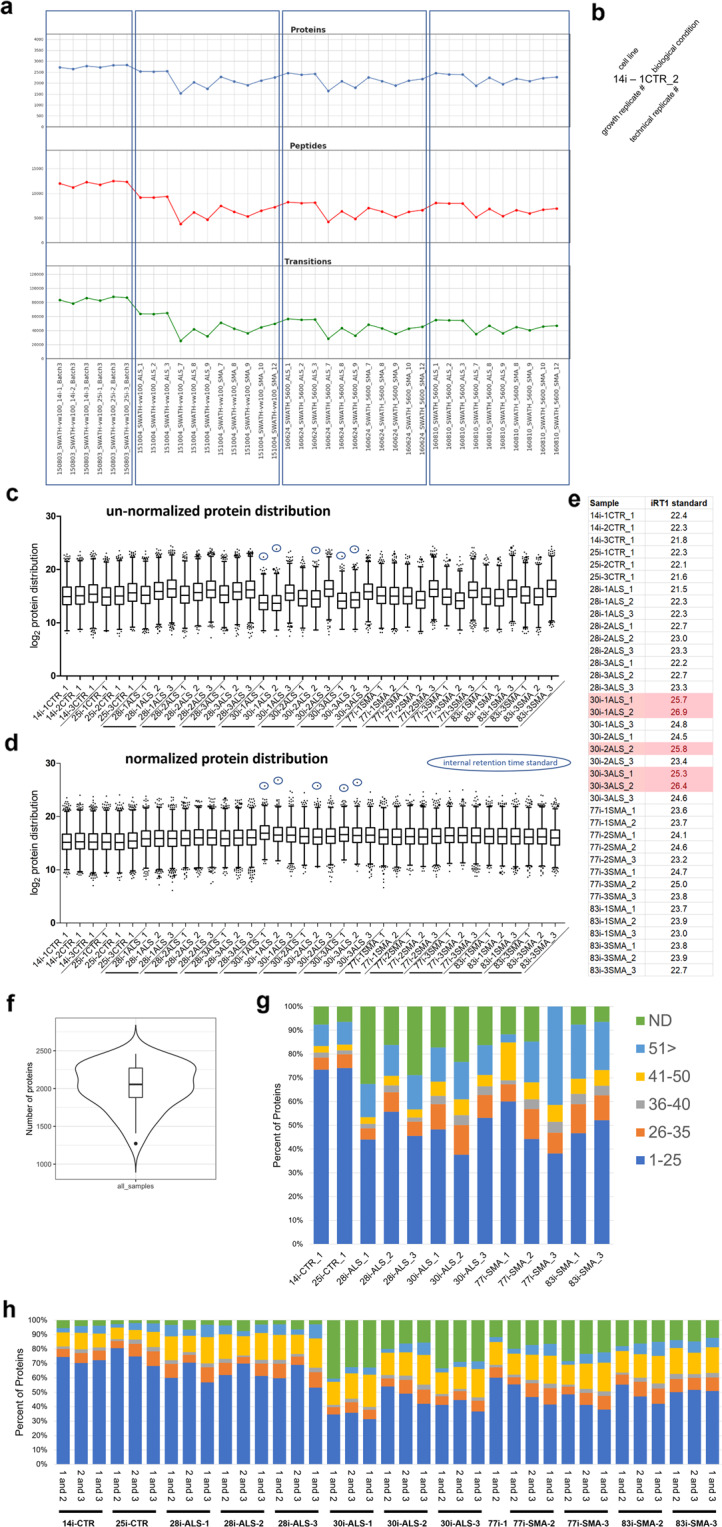
Data consistency across sample analyses. (**a**) Number of proteins, peptides and transitions identified per sample, including biological growth replicates and temporally dispersed technical replicates. MS raw file and sample metadata are available in the online Supplementary Table 4. (**b**) nomenclature key for data labeling, cell line, growth replicate, biological condition, and biological growth replicate. (**c**) distribution of raw un-normalized protein quantitation log_2_ signal intensity. (**d**) normalized protein concentrations were calculated by dividing each transition intensity by the sum of transitions measured for that sample. Outlier iRT protein data points (circled) in samples with higher iRT to total protein ratios. (**e**) log_2_ intensity of iRT per sample after normalization. iRT measurements greater than 25 are highlighted red. (**f**) statistics of proteins identified across all data files (n = 38). (**g**) distribution of protein coefficient of variation (CV) for biological replicates of each cell line. CV for all biological replicates, per bar, and technical replicates. (**h**) CVs calculated in pairs for each biological replicate. Calculated CVs provides resolution of data quality assessments.

For the DIA-MS data, the number of proteins and peptides quantitated using OpenSWATH provides the first tier of data curation (Fig. 2a). Quality data files that fall below a minimum number of quantifiable peptides, simply due to limited sample, adversely affect the extraction potential of equally loaded sample data files. Once these data files, limited by sample amount available for analysis are removed, the number of peptides quantitated is maximized for the remaining data files when searched simultaneously. Based on this criterion, samples that resulted in less than 2,000 quantifiable proteins in combination with the cell line sample quality assessment mentioned, two cell lines per experimental group, CTR, ALS and SMA, were removed from further analyses. This left 2 cell lines per experimental group for a total of 6 different patient-derived hiPSC lines. Once sample normalization was performed, the highest abundant protein data points were circled (Fig. 2c,d) as outliers. These data points were investigated further to determine what they were and if they were the same protein in each sample. Of note, these outliers were only in same that quantified the lowest number of proteins comparatively, albeit above 2,000 proteins as well as overall higher %CVs for the proteins quantified. All five circled data points were identified as the internal retention time standard, which gets spiked into all samples. These samples, where iRT peptides were the most abundant and more abundant than detected in any other sample (Fig. 2e) resulted from a higher ratio of iRT peptides to total protein and are, therefore, not considered a defect in the normalization method used.

To assess stability of the hiPSC culture protocol and consistency between growth plates, analysis of 3 biological growth replicates were performed for each cell line using different wells of the culture plate and thus grown simultaneously. The percent coefficient of variation (%CV) analyses were performed on the protein level data of 6 hiPSC lines collected in 3 biological replicates (Fig. 2g). To assess the stability of frozen, digested peptide samples and instrument conditions, 3 technical replicates were also generated from each of the ALS and SMA lines, both 8 and 10 months following the initial data acquisition. To determine if some of the samples were outliers, causing overall higher %CVs, each sample was replotted in pairs for %CV calculations (Fig. 2h). For the first technical replicate of cell line 28i-1ALS, (Fig. 2e), only ~40% of proteins between 3 biological replicates have a %CV less than 25. However, biological replicate one has the lowest correlation, i.e., high %CV when compared to either replicate 2 or 3. In Fig. 2f, 28i-1ALS, 70% of the proteins quantified in biological replicates 2 and 3 have a %CV less than 25. Therefore, biological replicate 1 of 28i was removed from biological analyses due to decreased sample quality based on low correlation with the remaining 2 biological replicates of the 28i-ALS cell line and thereby eliminates experimental variability that would otherwise cloud the biological interpretation of, in this case, ALS.

**Fig. 3:**
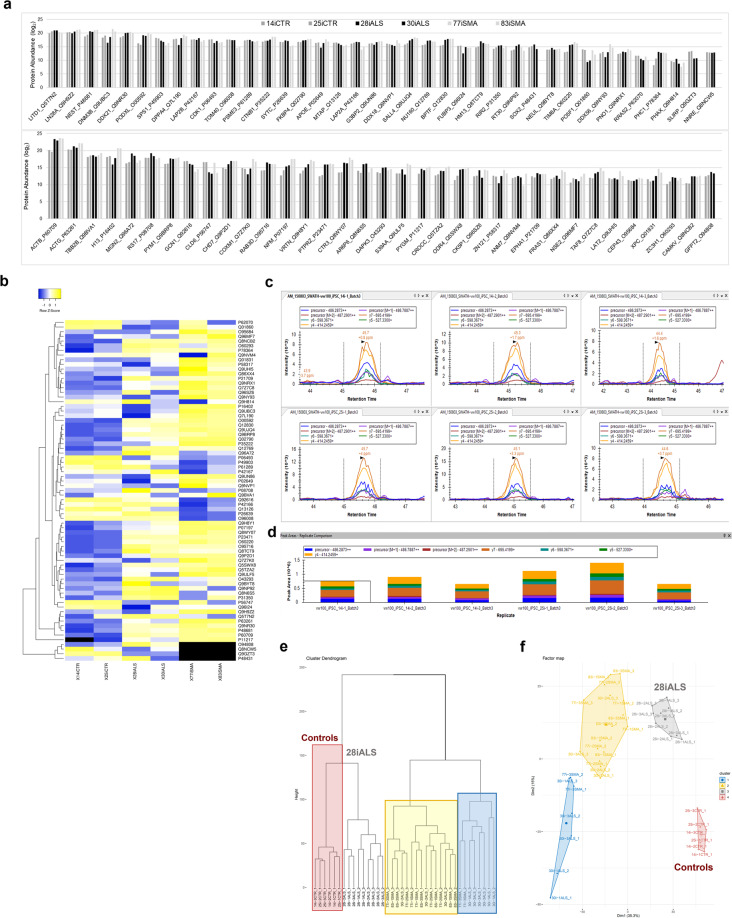
Reproducibly quantified protein markers of human iPSC lines. (**a**) 73 protein markers of pluripotency reproducibly quantified in 6 patient-derived iPSC lines. Protein quantitation was averaged across all growth and technical replicates for each cell line. (**b**) Heatmap of 73 protein hiPSC markers. (**c**) Extracted ion chromatograms (XICs) for peptide K.LYPAIPAAR.R [562, 570] of DNA (cytosine-5)-methyltransferase 3B in hiPSC lines 14i and 25i for three biological growth replicates. (**d**) Skyline peak area plot of K.LYPAIPAAR.R [562, 570] transition ions. (**e**) unbiased sample clustering of DIA-MS hiPSC samples and replicates by hierarchical clustering and (**f**) unbiased sample clustering by PCA.

Peptide signals were extracted to determine if known proteins routinely used to identify and characterize hiPSC cultures by immunofluorescent staining could be accurately and reproducibly quantitated in these DIA-MS analyses. A list of proteins used to characterize hiPSC cultures was generated in addition to proteins previously published on human iPSC samples^7,61,62^. In all, 73 proteins were extracted from the average 2300 proteins quantified (Fig. 3). Protein quantitation of these hiPSC markers were stable, showing minimal quantitative fluctuation across all 6 genetically diverse hiPSC lines analysed. This and other hiPSC proteomic studies may serve to further annotate protein databases of hiPSC protein expression and hiPSC biology in comparison to other human cell types or iPSCs originating from other organisms. Pripuzova, *et al*. published a panel of 22 candidate protein markers of hiPSCs from the analysis of 10 hiPSC lines. Expression of these protein were first discovered by LCMS in 2 hiPSC lines and later confirmed by WB in an additional 8 lines^62^. Of the 22 hiPSC proteins, 17 of these were able to be quantified in the panel of 73 hiPSC proteins produced in this study. StemCellDB^63^ (http://stemcelldb.nih.gov) used microarray global gene expression analysis to generate a gene list of 82 markers of which 30 of them are quantified on the protein level and are included in the panel of 73 hiPSC protein markers. Other embryonic proteins are included, such as Taf8, known for its role in early embryonic development albeit not previously characterized in hiPSCs to our knowledge. According to UniProt, Taf8 (by similarity) maybe important for survival of cells of the inner cell mass which constitute the pluripotent cell population of the early embryo.

Other proteins included in the panel of 73 are associated with aberrant expression in cancer cells. Cancer has been one of the most highly researched diseases. Though a hallmark of cancer is the cell’s ability to de-differentiate into a more pluripotent or stem cell-like state thus enabling increase cell replication and acquiring metastatic capabilities known as the epithelial-to-mesenchymal transition (EMT). Protein mapping to Ensemble transcript and CPTAC identifiers were compiled (online Supplementary Tables 2 and 3). From the CPTAC resource, 11 proteins overlap with the 73 iPSC proteins out of the 1464 proteins targeted in the various cancer studies made available (https://gdc.cancer.gov). As annotations of cellular proteomes are explored, more overlap between these cell types should be expected. Ultimately, 73 proteins representing biological signatures of human iPSCs (level 1) and separate tables of these proteins and peptide quantitation per cell line from all technical and biological replicates analysed are provided (data level 4).

After data quality assessments, the subset of hiPSC lines presented (38 MS raw files for 6 of 12 original lines) contain high quality protein and peptide data from which the 73 protein markers of human iPSCs are a valuable contribution to lists previously published^7,61,62^. Aside from 2 cell lines that were unusable due to mixed cell line contamination, as previously stated, it is worth noting that the entire workflow was improved for all future NeuroLINCS proteomics analyses of motor neuron cultures^58,59^. The hiPSC study being the first analysis in the development of the pipeline with ultimate goals of being conducive to high-throughput analyses required for motor neuron samples of NeuroLINCS and, later, for Answer ALS^59^. Therefore, the small samples of the cultured iPSCs were intended to reduce cost, however the methods used to process samples required improvements methods to minimize the sample loss associated with the workflow used for the hiPSC samples of this study, which is directly responsible for the limited protein depth reported for the hiPSCs.

Making this data publicly available does not come without a few words of caution^29–34^. An important consideration is that this hiPSC experiment was designed around understanding proteome variability rather than disease specific biology, in contrast to the experimental design of other NeuroLINCS data releases 2 and 3 (Table 1), for which inducible motor neuron cultures were analysed. Therefore, disease versus control analyses for this iPSC data set cannot and should not be performed because there is no way to tease apart the technical variability of the batch effects from true disease specific biology since sample processing and data acquisitions were performed separately for the control and disease sample sets. Any attempt to derive disease specific biological meaning from the apparent clustering in the dendrogram and PCA plot (Fig. 3e,f) for the purpose of disease specific biology, would be misguided. Instead, the PCA plot is a testament to the great quality of 2 patient control cell lines that tightly cluster and 28iALS biological replicates and 2 sets of individual technical replicates generated from frozen aliquots of the original sample digests, 8 and 10 months following the initial acquisition. Each cell line clusters tightly with its biological growth plate replicates and technical replicates. Therefore, sample clusters represent the quality of sample storage and technical reproducibility of the sample generation, sample processing and instrumentation. Continued development of both a semi-automated sample processing workflow and new DIA-MS methods have occurred since this initial iPSC experiment that deliver improved proteome coverage, depth and precision while requiring less sample. Automation and small sample requirements are essential to accomplishing large scale, population-based proteomic studies of the future. This proteomic data stands to understand the nuances hiPSC protein biology from cell lines of several human subjects and to further annotate hiPSC specific resources as the field continues to explore human proteome variability across individuals, from different cell and tissue types or altered experimental conditions.

## Usage Notes

Data level 0 – raw MS files.

- Compare detection and quantitation to other human cell lines or cell types

- Mine unidentified peptide spectra from data-dependent acquisition (DDA) files
- Extraction of peptide identifications from new or updated Uniprot fasta files or other protein sequence databases with isoform sequences or genetic variations resulting in peptide sequence changes.
- Raw data analysis using alternative data conversion and extraction algorithms.
- Bioinformatic development or vetting novel MS-proteomics algorithms or data mining tools.
- Test new or updated search algorithms and mass spectral data normalization across independent data sets^44,57^.
- DIA-MS analysis of co- and posttranslational modifications has challenges and is an evolving aspect to these complex data sets^28,59–63^.
  Data level 1 – Skyline documents of 73 quantified human iPSC proteins in 6 cell lines.
- Compare detection and quantitation to other human cell lines, cell types including cancer cells, oocyte and embryonic stem cells.
- Compare detection and quantitation to various states of hiPSC cultures^64^.
  Data level 2 – unnormalized protein and peptide levels.
- Protein expression values may be combined or compared with other hiPSC or neuronal datasets with careful considerations of signal correlations and overall compatibility of independent studies and normalization methods.
- Use to understand differences in detection based on technical methods used.
  Data level 3 – normalized protein and peptide levels.
- Mine protein and peptide expression profiles.
- Explore data with respect to available patient, cell line and experimental metadata.
- Data integration studies using NeuroLINCS transcriptomic and epigenomic data generated from aliquots of the same sample for transcriptomics data or from the same cellular stock for epigenomic data.
  Data level 4, signature – List of protein markers of pluripotency quantified in hiPSC samples. Signatures of differentiated motor neuron cultures of the same cell lines are available as fold change values of protein expression between disease and control cell lines.
- Determine coverage of hiPSC markers in hiPSC MS-based data sets. 
- Match differential signatures in other hiPSC or neuronal disease studies to find signature overlap on the peptide, protein or molecular pathway level^65,66^.

## Supplementary information

Online supplementary tables.

## Data Availability

Computer code used for data analyses of this manuscript are previously published and referenced in the Methods section.
